# Initial Potassium Replacement in Diabetic Ketoacidosis: The Unnoticed Area of Gap

**DOI:** 10.3389/fendo.2018.00109

**Published:** 2018-03-21

**Authors:** Atif Usman

**Affiliations:** ^1^School of Pharmacy, Monash University Malaysia, Bandar Sunway, Selangor, Malaysia

**Keywords:** diabetic ketoacidosis, hypokalemia, potassium replacement, treatment outcome, cardiovascular diseases, mortality

## Basics of Diabetic Ketoacidosis (DKA)

Diabetic ketoacidosis is an acute complication of diabetes mellitus (DM). It affects all the types of DM and hence is a continuous threat for all the diabetes patients (1). DKA is a well-studied disease. Among the precipitating causes, mostly reported factors are non-compliance of patients with the antidiabetic treatment, and infection; others, however, may not have any precipitating cause (1, 2). The progress of disease is very simple; lack of insulin causes hyperglycemia and inability of glucose to enter the cell. In-turn, triglycerides are broken down to free fatty acids which are used as a source of energy (1, 3, 4). In due process, the end-product of this metabolic derangement, i.e., ketones, cause acidification of blood causing major disruption in homeostasis. Similar to pathophysiology, the treatment of DKA is also simple and encompasses administration of insulin to achieve euglycaemia, and administration of crystalloid or colloidal solution to attain euvolaemia and euelectrolytaemia (1–3). Nevertheless, by the time patient reports for medical attention, these simple derangements and the rectification pathway have had gone significant derailment with potassium being the most affected ion throughout the event and resolution of DKA (5, 6). In similar context, potassium adds on to further exacerbation of already compensating, due to acidosis and respiratory distress observed in DKA, cardiovascular system (CVS).

## Loss of Potassium During Episode of DKA

Contrary to the simple pathophysiology, the combined effects of hyperglycemia and acidosis vastly impact major organ systems. Most of these effects are observed on CVS. First entity to get disturbed by the lack of insulin is electrolytes among which, potassium is most affected (6, 7). Apart from glucose, insulin is also responsible for the cellular regulation of potassium whereby it promotes the cellular influx of potassium from extracellular compartment (7–9). Moreover, stress induces insensitivity to cells, which are unable to uptake potassium *via* catecholamine-driven pathway (9, 10). Both these factors increase the serum concentration of potassium ions and on top of it, serum analysis may reveal a false hyperkalaemia as well (11, 12). On the other hand, hyperglycemia induced by lack of insulin promotes diuresis. Such procedure causes loss of water content from the body during which, sodium ion tends to retain itself at expense of excretion of potassium ion. Moreover, the renal loss of potassium is further observed with acidosis; hydrogen ion from the bicarbonate nucleus follows the same pattern as of sodium ion and is reabsorbed at the expense of potassium ion (13). In addition to the renal loss of potassium, gastrointestinal tract is also a major route *via* which, significant amount of potassium is lost. Ideally, body maintains osmotic pressure at expense of serum and tissue electrolyte. In case of acute hyperkalaemia, gastric cells preserve hydrogen ions’ concentration. This promotes epigastric distress, which in-turn induces vomiting and diarrhea thereby promoting the loss of potassium ion as well (1, 14). Loss of potassium, hence, has profound effect on the cardiac conduction, and, resultantly, on function as well.

## Recommendations for Potassium Resuscitation

As mentioned earlier, late-sought medical attention for DKA causes the treatment to be complex as well. Under normal conditions, i.e., when patient is not comatose, the patient is mostly started on either intravenous or basal bolus insulin, and normal saline. As per recommendation of various guidelines, the important biochemical profiles include analysis of blood gasses and renal profile. It is widely suggested that the normal saline shall be used for initial resuscitation and once the potassium level is retrieved, the patient can be started on potassium replacement should the serum potassium level be between 3.3 and 4.5 mmol/L (3, 15, 16). All the major guidelines further acknowledge that insulin mediated reentry of potassium from extracellular to intracellular compartment may precipitate hypokalemia, and hence, insulin should be withheld if the serum level of potassium is below 3.3 mmol/L. Although that the potassium level is given appropriate representation (Table 1), however, the extent of being deceived with the retrieved level of potassium may end up in a lethal outcome.

**Table 1:** Recommendation of potassium replacement by different guidelines.

Guideline	NICE (15)	Kitabchi et al. (3)	JBDS (16)	CPG Malaysia (17)
Recommended potassium replacement	(A) Use 20 mmol of K^+^ in each 500 mL fluid bag	(A) If urine output is ~50 mL/h and: (a) Serum K^+^ is below 3.3 mEq/L (i) Hold Insulin (ii) Give 20–30 mEq K^+^/h (b) Or serum K^+^ is above 5.3mEq/L (i) Omit K^+^ (c) Or serum K^+^ ~3.3–5.3 mEq/L (i) 20–30 mEq K^+^/L	(A) If K^+^ is: (a) Above 5.5 mmol/L; omit (b) Within 3.5–5.5 mmol/L; 40 mmol/L (c) Below 3.5 mmol/L; consult	(A) If K^+^ is: (a) Above 5.5 mmol/L; nill (b) Within 3.5–5.5 mmol/L; 40 mmol/L (c) Below 3.5 mmol/L; consult

## Unnoticed Area of Gap for Serum Potassium

The level of potassium may not have been accurate as per the actual retrieved value, and hence, patient may experience life threatening effects in form of ventricular fibrillation and ventricular tachycardia (14, 18). Applying the model proposed by Shargel in a fictitious scenario, a patient reporting to the hospital for DKA with a pH of 7.1 and potassium level of 3.5, may already have a life threatening level of potassium, i.e., 2.3 mmol/L (19). Strikingly, all the guidelines recommend to use the insulin and fluid resuscitation in the form of normal saline as soon as the patient is admitted so as to cease the production of ketones; none of these guidelines recommends to calculate the corrected level of potassium, and hence, the patient may be at risk of acquiring spectrum of acute hypokalaemic complications. To get actual spectrum of the risk for DKA patients, consider prevalence of CV diseases among diabetic patients and dysvolaemia-induced CVS and pulmonary edema risk.

Literature encompassing risk of hypokalemia induced-CVS outcomes in DKA patients is very low, if not scarce; although the citations from mid 1900s do have some reports stating it as a problem. Given example of Baker (20), the author recorded personal experience of admissions of DKA; debated then-know most important entities, i.e., insulin, use of normal saline, and use of alkali to resolve acidosis; presented the cases that had signified difference from the rest of reported patients; and debated and recommended appropriate use of insulin with personal experience. Interestingly, the role of potassium is neither discussed nor mentioned despite that five mortalities each was attributed to CVS and unknown cause. In addition to Baker, the researches carried around the same span of time were in context of experimentation with insulin and hence, the extent of CVS involvement with the serum potassium is either not clear or not given appropriate representation. As soon as Holler describes the extent of potassium involvement with the DKA and insulin, a few reports follow the trend with varying objectives despite the fact that the role of insulin on electrolyte profiles was being extensively studied in animals (21–25). Nevertheless, following couple of decades in fact focused on dose and route of insulin. Although improvement in the overall efficacy and safety of most of the guidelines being used today is well proven with years of research and practice, what are the cardiovascular impacts of lack of corrected-potassium induced replenishment in DKA protocols have yet to be established.

## Proposed Strategy

A systematic review or a meta-analysis to search for the association of CV outcomes of corrected serum potassium levels during diagnosis and management of DKA will surely help to establish a relationship of aforesaid two parameters. The systematic search can be carried of the literature that reports either retrospective or prospective, cohorts or case series, prevalence or incidence of DKA, or the clinical trials reporting efficacy of main components of DKA management that may affect serum concentration of potassium ion. In addition of mentioned criteria, by targeting only the studies reporting effects of potassium ions on CVS as a primary, secondary, or subsidiary outcome of the research will help to determine practicality of this opinion. Should there be the data to compile so as to establish a statistical association or dissociation, it will help to understand and modify all the major current guidelines and hence may improve the CVS outcomes for the DKA patients.

## Author Contributions

Idea, conceptualization for further research, and authorship are developed and undertaken by AU at School of Pharmacy, Monash University Malaysia.

## Conflict of Interest Statement

The author declares that the research was conducted in the absence of any commercial or financial relationships that could be construed as a potential conflict of interest. The handling editor declared a shared affiliation, though no other collaboration with the author AU.
